# Predictors of sun protection behaviours and sunburn among Australian adolescents

**DOI:** 10.1186/s12889-016-3197-4

**Published:** 2016-07-13

**Authors:** Simone Pettigrew, Michelle Jongenelis, Mark Strickland, Carolyn Minto, Terry Slevin, Geoffrey Jalleh, Chad Lin

**Affiliations:** School of Psychology and Speech Pathology, Curtin University, Perth, Western Australia; Cancer Council Western Australia, Perth, Western Australia; School of Public Health, Curtin University, Perth, Western Australia

**Keywords:** Sunburn, Sun protection, Tanning, Adolescents

## Abstract

**Background:**

Excessive sun exposure and sunburn increase individuals’ risk of skin cancer. It is especially important to prevent sunburn in childhood due to the higher relative risk of skin cancer across the life span compared to risk associated with sunburn episodes experienced later in life. This study examined demographic and attitudinal factors associated with engagement in a range of sun protection behaviours (wearing a hat, wearing protective clothing, staying in the shade, and staying indoors during the middle of the day) and the frequency of sunburn among Western Australian adolescents to provide insights of relevance for future sun protection campaigns.

**Methods:**

Cross-sectional telephone surveys were conducted annually with Western Australians between 2005/06 and 2014/15. The results from 4150 adolescents aged 14–17 years were used to conduct a path analysis of factors predicting various sun protection behaviours and sunburn.

**Results:**

Significant primary predictors of the sun protection behaviours included in the study were skin type (sun sensitivity), gender, tanning-related attitudes and behaviours, and perceived relevance of public service advertisements that advocate sun protection. Of the four sun protection behaviours investigated, staying in the shade and staying indoors during the middle of the day were associated with a lower frequency of sunburn.

**Conclusion:**

There is a particular need to target sun protection messages at adolescent males who are less likely to engage in the most effective sun protection behaviours and demonstrate an increased propensity to experience sunburn. The results suggest that such future sun protection messages should include a focus on the importance of staying in the shade or indoors during periods of high UV radiation to increase awareness of the efficacy of these methods of avoiding skin cancer.

## Background

Excess sun exposure and sunburn are primary causes of skin cancer [1, 2]. Skin cancer is one of the most commonly diagnosed forms of cancer [3], and incidence rates are especially high in countries such as Australia that are characterised by high levels of ultraviolet (UV) radiation [4, 5]. As skin cancer rates are continuing to increase around the world [5–8], more concerted efforts are required to encourage individuals to adopt lifestyle behaviours that minimise their risk by engaging in sun protection behaviours [6, 9, 10].

Substantial inroads have been made in Australia in reducing the attractiveness of tanned skin and encouraging individuals to adopt sun protection practices such as staying indoors during peak UV times and wearing sunscreen and protective clothing when in the sun [9–12]. However, improvements appear to have plateaued in recent years and many people continue to place themselves at risk on a regular basis by failing to take these basic precautions, especially those relating to wearing protective clothing, staying in the shade, and staying indoors during times of peak ultraviolet radiation [11]. Analyses of possible contributing factors have identified assumptions of a tan being healthy [13], representations of tanned bodies in the media [14], and, more recently, media coverage of the possibility of vitamin D deficiency [15] as being barriers to optimal engagement in sun protection behaviours. These factors may prevent individuals from appreciating the skin cancer risks associated with excess sun exposure, and understanding of risk is critical in motivating sun protection behaviours [16, 17].

Specific demographic characteristics have been associated with stronger preference for a tan, attempting to achieve a tan, and experiencing sunburn. Across different countries, younger people and females tend to report greater preference for and attempts to achieve a tan relative to their older, male counterparts [10, 18–21]. Pro-tanning attitudes and behaviours among young people are concerning given that exposure to UV radiation prior to reaching adulthood is recognised to be the most critical risk factor for the development of skin cancer over the life course [1, 22]. This makes young people an especially important target for public health campaigns that aim to promote sun protection behaviours [18, 23].

The development, implementation, and ongoing refinement of effective skin cancer prevention campaigns are reliant on a detailed understanding of community attitudes and behaviours in relation to sun protection and how these change over time. In particular, it is important to identify population segments that are responding well to current messages and those that remain resistant and may therefore require alternative messaging approaches. In Western Australia, the context of the present study, the SunSmart television campaign has been running since 1990. The aim of this campaign is to encourage people to reduce their sun exposure by engaging in sun protection behaviours promoted in the iconic ‘Slip Slop Slap’ advertisement that recommended ‘slipping’ on a shirt, ‘slopping’ on sunscreen, and ‘slapping’ on a hat [24]. Since its inception, the SunSmart campaign has featured a range of advertising themes and executions, but a consistent element across most years has been a focus on young people in recognition of their higher sunburn rates, stronger pro-tanning attitudes and behaviours, and their greater vulnerability to lifelong skin cancer risk.

The aim of the present study was to assess predictors of Western Australian adolescents’ (14–17 years) experience of sunburn and their enactment of key sun protection behaviours. Western Australia experiences high levels of ultraviolet radiation [25], making sun protection particularly important in this geographical location. The sun protection behaviours of interest were wearing hats and other protective clothing, staying in the shade, and staying indoors during times of peak UV radiation. Sunscreen use was not included due to previous research demonstrating that sunscreen use and endorsement is already at relatively high levels in Australia [11, 26], and hence the need to encourage other forms of sun protection that sit higher on the sun protection hierarchy [27, 28]. By identifying factors associated with specific forms of sun protection that remain under-utilised, the study results provide insights of direct relevance for future campaigns designed to reduce skin cancer risk in Australia and elsewhere.

## Methods

As part of ongoing monitoring of Western Australians’ sun protection behaviours, annual telephone surveys are conducted with adolescents and adults each summer. The surveys include items relating to tanning attitudes and behaviours and any experience of sunburn over the summer. The present study relates to the data collected from adolescents in the ten surveys conducted over the decade 2005/06 to 2014/15 (adult results reported elsewhere [11]).

Random digit dialling was used to access households throughout the state, with only one person per household eligible to complete the survey. Each year over the 10 year study period, between 299 and 611 adolescents aged 14 to 17 years completed the survey. Quotas were used to achieve an even gender and age spread. The final sample comprised 4150 adolescents (2073 male and 2077 female). Table 1 provides the demographic characteristics of the sample.

**Table 1:** Total Sample Profile

	Total	
	*N* = 4150	
Attribute	n	%
Gender		
*Male*	2073	50.0
*Female*	2077	50.0
Age (years)		
*14*	933	22.5
*15*	1155	27.8
*16*	1120	27.0
*17*	942	22.7
Socioeconomic status^a^		
*Low*	385	9.4
*Medium*	1929	47.2
*High*	1770	43.3
Skin colour		
*Very fair*	433	10.4
*Fair*	1338	32.2
*Medium*	1283	30.9
*Olive*	892	21.5
*Dark*	174	4.2
*Very dark*	14	0.3
*Black*	4	0.1
*Don’t know*	12	0.3
Sun sensitivity		
*Just burn, no tan*	840	20.2
*Burn, then tan*	1959	47.2
*No burn, just tan*	1190	28.7
*Nothing*	132	3.2
*Can’t say*	29	0.7

^a^Missing values not included (*n* = 66; 1.6 %)

### Measures

The demographic data captured included age, gender, and postcode (for socioeconomic status estimation as per the Australian Bureau of Statistics’ Socio-Economic Indexes for Areas (SEIFA) [29]). Respondents were also asked to report their skin colour without a tan (seven response options ranging from ‘very fair’ to ‘black’) and their sun sensitivity (i.e., likelihood of burning) when in strong sunshine for 30+ minutes (four response options: ‘nothing would happen’, ‘not burn at all, just tan’, ‘burn first, then tan afterwards’, and ‘just burn and not tan afterwards’).

To assess sunburn frequency, respondents were asked how often they had been sunburnt over the most recent summer. The four response options were ‘never’, ‘once’, ‘2 to 3 times’, and ‘4 or more times’. Tanning-related attitudes and behaviours were investigated via items asking respondents whether they liked to suntan (yes/no dichotomous response option) and whether they had attempted to tan during the most recent summer (yes/no dichotomous response option). Respondents were also asked how frequently they would engage in the following sun protection behaviours when in the sun for an hour or more during the middle of the day in summer: wearing a hat, wearing clothes that cover most of the body, staying mainly in the shade, and spending most of the time inside. The five response options ranged from ‘never’ to ‘always’. Finally, those respondents indicating they recalled seeing a SunSmart sun protection advertisement during the previous summer were asked the extent to which they considered the advertisement to be personally relevant. The four response options ranged from ‘completely irrelevant’ to ‘very relevant’.

### Analysis

Data across survey years were pooled for analysis, with year of survey used as an independent variable in regression analyses to assess for any differences over time in the dependent variables of engagement in protective behaviours and sunburn frequency. Multiple linear regression analyses were conducted to investigate the factors associated with engagement in each of the sun protective behaviours and sunburn frequency. Regression analyses were accomplished in two steps. Univariate analyses were initially conducted in SPSS to assess the significant contribution of each independent variable without the influence of multicollinearity. Significant factors were then combined and included in a multivariate regression model that was assessed using structural equation modelling techniques in M*Plus* 7.2.

## Results

### Descriptive statistics

Sun-related attitudes and behaviours for the total sample are presented in Table 2. Two-thirds (66 %) of respondents reported being sunburnt at least once in the most recent summer and 31 % reported actively attempting to tan. The most frequently reported sun protection behaviours were staying in the shade and staying mostly inside, with 41 and 40 % of respondents respectively engaging in these behaviours ‘usually’ or ‘always’.

**Table 2:** Descriptive statistics for non-demographic variables under investigation

Variable	Response	n	%
Sunburnt this summer	Never	1396	33.6
	Once	942	22.7
	2 or 3 times	1408	33.9
	4 or more times	404	9.7
Like a tan	Yes	1970	47.5
	No	2180	52.5
Attempted to tan	Yes	1283	30.9
	No	2867	69.1
Engagement in protective behaviours			
Wearing a hat	Never	877	21.1
	Rarely	871	21.0
	Sometimes	1073	25.9
	Usually	884	21.3
	Always	445	10.7
Protective clothing	Never	863	20.8
	Rarely	1124	27.1
	Sometimes	1030	24.8
	Usually	880	21.2
	Always	253	6.1
Staying in the shade	Never	76	1.8
	Rarely	477	11.5
	Sometimes	1889	45.5
	Usually	1516	36.5
	Always	192	4.6
Staying mostly inside	Never	91	92.2
	Rarely	650	15.7
	Sometimes	1753	42.2
	Usually	1497	36.1
	Always	159	3.8

### Factors associated with frequency of engagement in sun protection behaviours and sunburn

The following were entered as independent variables in univariate regression analyses assessing the factors associated with engagement in the four sun protection behaviours: gender (0 = male, 1 = female), age, location (0 = metro, 1 = country/regional), socioeconomic status (SES), year of survey, sun sensitivity, attitude to tanning (0 = does not like to tan, 1 = likes to tan), attempt to tan (0 = no attempt to tan, 1 = attempt to tan), and perceived relevance of the SunSmart sun protection campaigns run during the study period. The same factors were also entered as independent variables in univariate regression analyses assessing predictors of sunburn frequency, with the addition of frequency of enactment of the four sun protection behaviours.

**Fig. 1:**
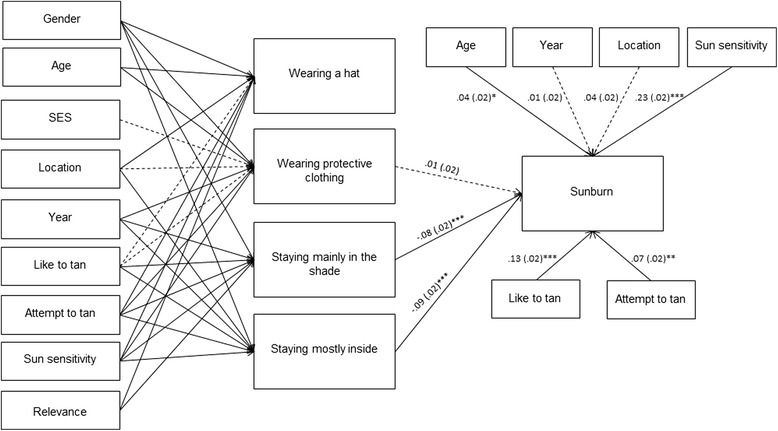
Model showing standardised regression coefficients with standard errors for factors impacting on sun protection behaviours and sunburn. *p < .05. ** *p <* .01. ***p < .001

When the significant variables identified in the multiple regression analysis were combined into a multivariate regression model and assessed in MPlus, a converged, admissible solution was obtained. The overall model (see Fig. 1) was a good fit to the data with a non-significant model chi square (*χ*2 (12) = 12.97, p = .371). Other fit indices met the criteria outlined for an excellent fit to the data: CFI = .999, TLI = .997, WRMR = .443, RMSEA = .005 (90 % CI: .000, .019; *p*-close = 1.00).

Given the inability to include all relevant path values in Fig. 1 due to the complexity of the model, the standardized parameter estimates for the variables purported to influence engagement in each of the protective behaviours are presented in Table 3. Gender was significantly associated with engagement in all four behaviours (all *p <* .001). Relative to males, females engaged less frequently in wearing a hat and protective clothing but stayed in the shade or inside more frequently. Age also emerged as significant, with younger adolescents wearing a hat and protective clothing more frequently than older adolescents. Those with greater sun sensitivity engaged significantly more frequently in all four protective behaviours.

**Table 3:** Standardised Parameter Estimates and Standard Errors for the Factors Associated with Engagement in Protective Behaviours

Independent variable	Dependent variable	*b*	*SE*	*b/SE*	*p* value
Gender	Frequency–hat	−0.21	0.02	−11.94	< .001
Attempt to tan	Frequency–hat	−0.15	0.02	−7.11	< .001
Age	Frequency–hat	−0.10	0.02	−5.96	< .001
Perceived relevance	Frequency–hat	0.10	0.02	5.88	< .001
Sun sensitivity	Frequency–hat	0.09	0.02	5.22	< .001
Location	Frequency–hat	0.07	0.02	3.91	< .001
Like to tan	Frequency–hat	−0.00	0.02	−0.19	.848
Attempt to tan	Frequency–clothing	−0.17	0.02	−7.88	< .001
Sun sensitivity	Frequency–clothing	0.10	0.02	6.01	< .001
Year	Frequency–clothing	−0.07	0.02	−3.96	< .001
Gender	Frequency–clothing	−0.07	0.02	−3.90	< .001
Age	Frequency–clothing	−0.05	0.02	−2.89	.004
SES	Frequency–clothing	−0.03	0.02	−1.62	.106
Like to tan	Frequency–clothing	−0.02	0.02	−0.91	.361
Location	Frequency–clothing	0.01	0.02	0.37	.714
Gender	Frequency–shade	0.16	0.02	8.97	< .001
Like to tan	Frequency–shade	−0.17	0.02	−7.79	< .001
Year	Frequency–shade	0.13	0.02	7.72	< .001
Attempt to tan	Frequency–shade	−0.15	0.02	−6.70	< .001
Sun sensitivity	Frequency–shade	0.06	0.02	3.36	< .001
Perceived relevance	Frequency–shade	0.04	0.02	2.39	.017
Like to tan	Frequency–inside	−0.17	0.02	−7.85	< .001
Gender	Frequency–inside	0.11	0.02	5.81	< .001
Attempt to tan	Frequency–inside	−0.09	0.02	−4.15	< .001
Year	Frequency–inside	0.07	0.02	4.00	< .001
Location	Frequency–inside	−0.06	0.02	−3.25	< .001
Sun sensitivity	Frequency–inside	0.04	0.02	2.01	.044

Liking a tan and attempting to tan both demonstrated a significant negative relationship with seeking shade and staying inside. Over time, frequency of wearing protective clothing decreased while frequency of seeking shade and staying inside increased. Finally, perceived relevance of sun protection campaigns was significantly associated with engagement in hat use and staying in the shade, with those perceiving greater relevance engaging in these behaviours more frequently.

Standardized parameter estimates for the variables purported to influence sunburn frequency are presented in Fig. 1. Inspection of these estimates revealed that sunburn frequency was significantly influenced by age, sun sensitivity, liking a tan, attempting to tan, and frequency of seeking shade and staying inside. Older adolescents, those with greater sun sensitivity, those liking a tan, and those attempting to tan were more likely to report increased frequency of sunburn. Those who more frequently engaged in the protective behaviours of seeking shade and staying inside were less likely to have been sunburnt.

## Discussion

The large sample used in the present study enabled a path analysis to be conducted of the factors associated with Western Australian adolescents’ enactment of various sun protection behaviours and the relationship between these behaviours and frequency of sunburn. The focus on adolescents reflects the higher levels of life time risk associated with sun exposure early in life [1, 22]. The model was an excellent fit to the data, indicating that the results are useful in highlighting the importance of targeting specific youth segments and emphasising particular forms of sun protection in future skin cancer prevention campaigns. However, the effect sizes for each of the predictors of the focal sun protection behaviours and sunburn were generally modest, which is likely to be at least partially attributable to the strong influence of situational and normative factors that are beyond the scope of this analysis [30, 31]. For example, preference for certain pastimes (such as outdoor sports or spending time at the beach versus participating in screen-based activities) will play a large role in determining sun exposure and therefore the potential to enact sun protection behaviours and experience sunburn [6, 32].

Of the four sun protection behaviours included in the study, staying in the shade and staying inside were associated with reduced frequency of sunburn. This finding provides support for interventions that aim to prevent sun exposure at peak ultraviolet radiation times and encourage children to participate in physical activity at other times of the day or indoors [33]. It also adds to the limited body of research providing insights into the relative effectiveness of different methods of reducing excess exposure to ultraviolet radiation (i.e., the sun protection hierarchy). Previous work has suggested that the most effective methods during peak times of the day are, in order of relative efficacy: staying indoors, seeking shade, wearing protective clothing (long-sleeved clothing and headwear), and using sunscreen [28, 34–36]. The findings of the present study suggest that only staying in the shade and staying indoors are (negatively) associated with frequency of sunburn for this adolescent sample. This may provide insight into previous research with Australian adults that has found a plateauing over time in sunburn rates and the avoidance of the peak radiation exposure by staying indoors or in the shade [11, 37]. It thus appears that for both adolescents and adults, a focus of sun protection interventions should be on minimising sun exposure in the middle of the day through the use of shade or staying indoors.

The demographic attributes that were most strongly associated with the sun protection behaviours under investigation were sun sensitivity and gender. Consistent with previous research in other countries, those with more sun-sensitive skin types were more likely to engage in all four protective behaviours [38, 39]. However, many people inaccurately assess their skin type [40], and the association between skin type and skin cancer risk only weakens (i.e., does not disappear) with darker skin type [1]. There thus remains a need to ensure those with lower levels of perceived sun sensitivity also appreciate the benefits associated with avoiding sun exposure in the middle of the day and do not self-exempt themselves from sun protection messages.

The results for gender were mixed, with females more likely to avoid sun exposure altogether by seeking shade or staying inside and males more likely to adopt behaviours that involve staying in the sun but wearing protective clothing. This pattern of protective behaviour was reflected in higher rates of sunburn among males due to their lower enactment of those forms of protection that predicted lower rates of sunburn (i.e., staying in the shade or indoors). Males may be engaged in more active pursuits that are conducted outdoors [41], and therefore require additional assistance to manage their sun exposure while doing so. Effective assistance is likely to be structural in nature given the need to stay in the shade or indoors because of the relative lack of effectiveness of protective clothing and previous research demonstrating the limitations of sunscreen use in preventing sunburn [42, 43]. The structural strategies may include scheduling processes that allocate outdoor events to times of the day with low UV radiation or the provision of shade or indoor options for these events.

The model indicates that attitudes to tanning and attempts to tan are also significant predictors of experiencing sunburn. Although almost half of the sample reported liking a tan (48 %), just less than a third (31 %) had attempted to get a tan in the most recent summer. This difference in preference versus behaviour, along with demonstrated reductions in both variables over time in previous Australian research with adolescents [10], suggests that sun protection media campaigns over recent decades have been successful in changing tanning-related behaviours such that young people are choosing to avoid tanning despite finding tanned skin attractive.

The role of sun protection campaigns was also evident in the relationship between perceived relevance of sun protection advertisements and two of the sun protection behaviours included in the study: wearing a hat and staying in the shade. To date, Western Australian SunSmart media campaigns have not generally encouraged wearing clothing that fully covers the arms and legs or staying indoors during peak UV times, which accounts for the lack of significance of these variables in the model. The results of the present study suggest that future campaign executions could be designed around reinforcing efforts to find shade when outdoors and recommending the scheduling of weekend outdoor activities during non-peak UV times to focus on those sun protection behaviours that were found to be associated with less frequent sunburn. The apparent effectiveness of prior campaigns in influencing hat wearing and shade seeking indicate that this approach has the potential to make meaningful improvements in sunburn rates among adolescents.

### Limitations and future research directions

This study has several limitations that can be addressed in future research. In the first instance, the sample is limited to Western Australians aged 14-17 years, and as such the model may have limited generalisability to other populations. Future research could assess the extent to which the relationships hold for those residing in other regions with different levels of UV radiation and different cultural norms in relation to tanning. Second, the use of dichotomous response options for tanning-related attitudes and behaviours is suboptimal, and future studies could offer respondents a greater number of response options to permit a more comprehensive analysis of the role of these important predictors of sunburn. Third, the analysis did not account for parts of the body that are burned and the severity of burning. It is highly likely that those wearing hats and long clothing would experience less extensive and less intense sunburn than those who do not take these precautions, but the present analysis was not sensitive enough to capture these differences. Future research may include these variables to provide a more complete understanding of the value of different sun protection measures. Similarly, many other variables are likely to also impact sun exposure behaviours, such as perceived risk of skin cancer and self-efficacy for avoiding skin cancer [44]. In addition, concerns in the community about vitamin D deficiency [45] may be impeding improvements in sun protection behaviours. The present model is already complex (see Fig. 1), which precluded the inclusion of these additional variables. However, the identification of some variables as non-significant will allow future models to potentially exclude these variables and replace them with other possible predictors to develop more comprehensive accounts of factors contributing to individuals’ experiences of sunburn.

## Conclusion

The results provide important insights into modifiable factors that could be addressed in future interventions designed to reduce skin cancer risk among young people. Consistent with previous research, there is an identified need to target sun protection messages at adolescent males who demonstrate an increased propensity to experience sunburn and are less likely to engage in the most effective sun protection behaviours. The relative lack of effectiveness of wearing a hat and long-sleeved clothing suggests that future sun protection messages should focus on staying in the shade or indoors during periods of high UV radiation, which may in turn require changes to the way in which sporting and other outdoor activities are scheduled. Ongoing efforts are also needed to continue to alter perceptions of the attractiveness of a tan and to discourage tanning behaviours among young people.

## Abbreviations

CATI, Computer Assisted Telephone Interviewing; CI, confidence interval; OR, odds ratio; SES, socioeconomic status; UV, ultraviolet.
